# The relationship between religious health fatalism, medication adherence, and health literacy among patients with hypertension: a cross-sectional study

**DOI:** 10.1590/1980-220X-REEUSP-2025-0494en

**Published:** 2026-08-07

**Authors:** Tanrıverdi Ömer, Gültekin Abdurrezzak

**Affiliations:** 1 Mardin Artuklu University, Faculty of Health Sciences, Department of Nursing, Mardin, Türkiye.; 2 Kahramanmaraş Sütçü İmam University, Afşin School of Health, Department of Nursing, Kahramanmaraş, Türkiye.

**Keywords:** Hypertension, Health Literacy, Medication Adherence, Nursing, Hipertensão, Letramento em Saúde, Adesão ao Tratamento Medicamentoso, Enfermagem

## Abstract

**Objectives:**

Hypertension remains a leading global public health challenge requiring consistent self-management, particularly through medication adherence and adequate health literacy. This study aimed to examine the relationship between religious health fatalism, medication adherence, and health literacy among patients with hypertension.

**Methods:**

A descriptive, cross-sectional study was conducted among 211 hypertensive patients in two public hospitals in southeastern Türkiye. Data were collected using a sociodemographic questionnaire, the Religious Health Fatalism Scale (RHFS), the Medication Adherence Report Scale (MARS), and the Health Literacy Scale (HLS).

**Results:**

The mean RHFS score was 59.41 ± 12.14, indicating a moderate-to-high level of fatalistic belief. Participants also demonstrated moderate levels of health literacy and medication adherence. A weak positive correlation was observed between religious health fatalism and health literacy (r = 0.16, p = 0.04).

**Conclusions:**

Although health literacy was positively associated with medication adherence in correlation analysis, this association did not remain statistically significant in the regression model. Religious health fatalism showed a stronger association with medication adherence.

## RESUMO

**Objetivos:**

A hipertensão continua sendo um dos principais desafios globais de saúde pública, exigindo autogerenciamento contínuo, especialmente por meio da adesão ao tratamento medicamentoso e de níveis adequados de letramento em saúde. Este estudo teve como objetivo examinar a relação entre o fatalismo religioso em saúde, a adesão ao tratamento medicamentoso e o letramento em saúde em pacientes com hipertensão.

**Métodos:**

Foi realizado um estudo descritivo e transversal com 211 pacientes hipertensos atendidos em dois hospitais públicos do sudeste da Turquia. Os dados foram coletados por meio de um questionário sociodemográfico, da Escala de Fatalismo Religioso em Saúde (RHFS), da Escala de Relato de Adesão à Medicação (MARS) e da Escala de Letramento em Saúde (HLS).

**Resultados:**

A pontuação média da RHFS foi de 59,41 ± 12,14, indicando um nível moderado de crenças fatalistas. O fatalismo religioso em saúde apresentou correlação negativa com a adesão ao tratamento medicamentoso (r = −0,21; p < 0,001). Foram observadas correlações positivas fracas entre fatalismo e letramento em saúde (r = 0,16; p = 0,04) e entre letramento em saúde e adesão ao tratamento medicamentoso (r = 0,17; p = 0,015).

**Conclusão:**

Embora o letramento em saúde tenha apresentado associação positiva com a adesão ao tratamento medicamentoso na análise de correlação, essa associação não permaneceu estatisticamente significativa no modelo de regressão. O fatalismo religioso em saúde apresentou uma associação mais forte com a adesão ao tratamento medicamentoso.

## INTRODUCTION

Hypertension (HT) is one of the most prevalent chronic conditions worldwide and remains a major public health concern despite being largely preventable and treatable^(1)^. According to the World Health Organization, approximately 1.28 billion adults aged 30–79 years are affected by hypertension globally, with nearly two-thirds living in low- and middle-income countries^(2)^. The burden of hypertension differs across income levels, with higher prevalence and lower rates of awareness, treatment, and control observed in low- and middle-income settings compared to high-income countries^(2)^. These disparities are often associated with differences in healthcare access, education, and socioeconomic conditions. In Türkiye, hypertension represents a significant public health problem, with national population-based studies reporting a prevalence of approximately 30–32% among adults^(3,4)^.

A major challenge in hypertension management is poor adherence to prescribed treatment regimens. Studies indicate that nearly half of individuals diagnosed with hypertension discontinue treatment within the first year, and among those who continue, only about half adhere consistently to their medication as recommended^(5)^. Multiple factors contribute to non-adherence, including limited knowledge, medication side effects, treatment costs, forgetfulness, and the chronic nature of therapy^(6)^.

Additionally, the asymptomatic nature of hypertension and patients’ beliefs regarding the necessity of medication may further reduce adherence. In the context of chronic diseases, individuals often rely on religious beliefs as a coping mechanism alongside biomedical treatment^(7)^. Health fatalism, defined as the belief that health outcomes are predetermined and beyond individual control, has been identified as an important factor influencing health behaviors^(8)^. Previous studies have shown that higher levels of fatalistic beliefs are associated with reduced engagement in health-promoting behaviors and treatment adherence^(8,9)^, and may also be related to lower levels of education and health literacy^(10)^. Religious and cultural factors play a significant role in shaping fatalistic attitudes. In some contexts, religious interpretations may reinforce the perception that illness is inevitable or divinely predetermined, which can reduce individuals’ perceived control over their health^(11)^. Furthermore, fatalism has been associated with health literacy. Individuals with limited health literacy may be more likely to adopt fatalistic beliefs due to difficulties in understanding disease processes and treatment requirements^(10)^. This interaction between fatalism and health literacy may negatively influence treatment adherence and overall health outcomes.

Although previous studies have examined the relationships between fatalistic beliefs, health literacy, and medication adherence, these variables have generally been investigated separately^(8,9,12,13)^. Evidence suggests that fatalism is associated with health behaviors and self-care^(8,9)^, while health literacy has been linked to treatment adherence and health outcomes^(13)^. However, limited research has explored how religious health fatalism, health literacy, and medication adherence interact simultaneously within the same population, particularly in culturally and religiously influenced contexts. Furthermore, the potential role of health literacy in shaping the relationship between fatalism and adherence remains unclear. Addressing this gap is important for developing culturally sensitive and effective interventions aimed at improving chronic disease management. Therefore, this study aims to examine the relationship between religious health fatalism, medication adherence, and health literacy among patients with hypertension.

These findings may have important implications for healthcare systems and nursing practice. Understanding the role of religious health fatalism in shaping patients’ health behaviors can support the development of culturally sensitive care models. Previous studies have emphasized that culturally tailored interventions are effective in improving hypertension management and patient engagement across diverse populations^(14)^. In healthcare settings, integrating the assessment of fatalistic beliefs into routine patient evaluations may help identify individuals at risk for poor medication adherence. From a nursing perspective, these findings highlight the critical role of nurses in delivering individualized education, enhancing health literacy, and addressing belief systems that may hinder effective self-management. Nurses contribute to improving health literacy by simplifying complex medical information, supporting patients’ understanding of treatment regimens, and promoting informed decision-making^(13)^. Through these roles, nurses can help patients better interpret health information and engage more actively in their treatment process^(14,15)^.

Therefore, this study aimed to examine the relationship between religious health fatalism, medication adherence, and health literacy among patients with hypertension.

This study tested the following hypotheses:

H1: Religious health fatalism is expected to be negatively associated with medication adherence.
H2: Religious health fatalism is expected to be negatively associated with health literacy.
H3: Health literacy is expected to be positively associated with medication adherence.

### Rationale

Previous studies suggest that fatalistic beliefs may reduce individuals’ engagement in disease self-management behaviors, including medication adherence. In addition, limited health literacy may contribute to fatalistic thinking and negatively influence adherence to treatment. Based on this framework, the present study examined the relationships among religious health fatalism, health literacy, and medication adherence in patients with hypertension.

## METHOD

### Research Design

This descriptive cross-sectional study was conducted among patients with hypertension.

### Study Setting

The study was conducted among patients with hypertension attending the internal medicine outpatient clinics of two public hospitals in Mardin, southeastern Türkiye, between December 2024 and March 2025. Patients who met the eligibility criteria and provided informed consent were included in the study.

### Population and Sample of The Study

The study included patients with hypertension who presented to internal medicine outpatient clinics between December 2024 and March 2025. An a priori power analysis was conducted using G*Power (version 3.1.9.4) to determine the required sample size. Based on an effect size of 0.30, a significance level of 0.05, and a statistical power of 0.95, the minimum required sample size was calculated as 138 participants. However, the study was ultimately completed with a total of 211 participants.

### Inclusion and Exclusion Criteria

The inclusion criteria were as follows: patients diagnosed with hypertension for at least 6 months, aged 18 years or older, without a history of psychiatric illness, and without any visual, auditory, or speech impairment that could hinder communication. Participants were recruited from patients attending the internal medicine outpatient clinics during the study period using a consecutive sampling approach. Eligible individuals were informed about the study, and those who agreed to participate were included after providing written informed consent. Patients who did not meet the inclusion criteria, declined to participate, or had incomplete or missing questionnaire data were excluded from the study.

### Sociodemographic Form

The sociodemographic data form included 10 items covering age, diagnosis, gender, marital status, educational level, cohabitation status, income level, place of residence, smoking status, and family history of hypertension.

**Religious Health Fatalism Scale (RHFS):** The scale was developed by Franklin et al.^(16)^ to determine whether health fatalism in general is related to health behaviours. The Turkish validity and reliability study of the scale was conducted by Bobov and Capik in 2020^(11)^. The scale consists of 17 items and one sub-dimension. The scale is in 5-point Likert type and the total score range varies between 17–85. As the score obtained from the scale increases, the level of fatalism tendency increases. In the original scale, Cronbach’s alpha reliability coefficient was found to be 0.91^(11)^. In the current study, Cronbach’s alpha reliability coefficient was found to be 0.91.

**Medication Adherence Reporting Scale (MARS):** The Medication Adherence Report Scale (MARS) is a widely used instrument for assessing medication adherence. The Turkish version of the scale was translated and validated by Temeloğlu Şen et al.^(10)^ in 2019.^(10)^ MARS consists of 5 items and is rated on a 5-point Likert scale ranging from 1 (always) to 5 (never). The total score is calculated by summing the item scores, yielding a possible range of 5 to 25. Higher scores indicate better medication adherence, whereas lower scores reflect poorer adherence. The internal consistency of the Turkish version was reported as acceptable, with a Cronbach’s alpha of 0.78^(10)^. In the present study, the Cronbach’s alpha reliability coefficient was calculated as 0.72.

**Health Literacy Scale (HLS):** The validity and reliability study for the Turkish adaptation of the scale originally developed by Suka et al.^(17)^ was conducted by Türkoğlu and Kılıç in 2021^(18)^. The scale consists of 14 items, each rated on a five-point Likert scale ranging from 1 (strongly disagree) to 5 (strongly agree), with total scores ranging from 14 to 70. Higher scores indicate higher levels of health literacy. The scale comprises three subdimensions: Functional Health Literacy (items 1–5), Interactive Health Literacy (items 6–10), and Critical Health Literacy (items 11–14). The Cronbach’s alpha coefficient of the original Turkish version was reported as 0.85. In the present study, the Cronbach’s alpha reliability coefficient was calculated as 0.78.

### Statistical Analysis

Data were analyzed using IBM SPSS version 23. Descriptive statistics, including number, percentage, minimum, maximum, mean, and standard deviation, were used to summarize the data. Normality of continuous variables was assessed using the Kolmogorov–Smirnov test. Since the scale scores did not show normal distribution, Spearman’s rho correlation analysis was performed to examine relationships between variables. In addition, simple linear regression analyses were conducted to explore the associations between religious health fatalism, medication adherence, and health literacy. Prior to regression analyses, assumptions including linearity, independence of errors, homoscedasticity, and normality of residuals were evaluated and found to be satisfactory. Statistical significance was set at p < 0.05.

### Potential Sources of Bias

Selection bias may have occurred because participants were recruited from two public hospitals located in the same geographical region of southeastern Türkiye, which may limit the generalizability of the findings to broader populations.

### Ethical Approval

Ethical approval was obtained from the Mardin Artuklu University Non-Interventional Ethics Committee (Decision No: 2024/11-23). Permission to conduct the study was also obtained from Mardin Training and Research Hospital. All procedures were conducted in accordance with the Declaration of Helsinki. Written informed consent was obtained from all participants prior to their inclusion in the study.

## RESULTS

The participants had a mean age of 59.86 ± 13.81 years, the duration of hypertension (years) was 8.99 ± 6.96, 58.3% were female, 89.1% were married, 43.1% lived in a provincial center, 39.82% were illiterate, 44.5% had income equal to expenditure, 66.4% had a family member with hypertension, and 54.5% were non smokers (Table 1).

**Table 1:** Sociodemographic characteristics of participants – Mardin, Türkiye, 2025 (n = 211).

	Mean ± SD	(min–max)
Age	59.86 ± 13.81	(24–100)
Duration of hypertension (years)	8.99 ± 6.96	(1–32)
	**Frequency**	**Percent**
**Gender**		
Male	88	41.7
Female	123	58.3
**Marital Status**		
Married	188	89.1
Single	23	10.9
**Education Status**		
Illiterate	84	39.8
Literate	38	18.0
Primary Education Graduate	50	23.7
High School Graduate	25	11.8
High School and University Graduate	14	6.6
**Living status**		
Alone	25	11.8
With spouse	46	21.8
With spouse and children	135	64.0
With relatives	5	2.4
**Income Status**		
Income Less than Expenditure	92	43.6
My income is equal to my expenses	94	44.5
My Income Exceeds My Expenditure	25	11.8
**Place of residence**		
Province	91	43.1
District	83	39.3
Village	37	17.6
**Cigarette Use**		
Yes	96	45.5
No	115	54.5
**Hypertension in the family**		
Yes	140	66.4
No	71	33.6

(Mean: Average; SD: Standard Deviation; Min: Minimum; Max: Maximum).

Overall, participants demonstrated moderate levels of health literacy and medication adherence, whereas religious health fatalism scores were comparatively high. Among the health literacy subdimensions, interactive and functional health literacy were similar and slightly higher, while critical health literacy had the lowest mean score, indicating comparatively weaker performance in this domain (Table 2).

**Table 2:** Descriptive statistics of religious health fatalism, medication adherence, and health literacy scores – Mardin, Türkiye (n = 211).

	Mean ± SD	(min–max)
Religious health fatalism score	59.41 ± 12.14	(21–85)
Medication adherence score	22.22 ± 3.27	(10–25)
Health literacy score	50.36 ± 8.10	(27–70)
Functional Health Literacy	17.58 ± 5.19	(5–25)
Interactive Health Literacy	17.90 ± 5.08	(5–25)
Critical Health Literacy	14.87 ± 3.49	(4–20)

Correlation analysis revealed several significant relationships among the variables. Age showed a moderate positive association with duration of illness and number of children, indicating that older individuals tended to have longer disease duration and larger families. The number of medications used was negatively associated with health literacy, suggesting that individuals with higher health literacy tended to use fewer medications. A weak positive relationship was observed between number of medications and number of children. Medication adherence showed no significant relationship with most variables; however, it was negatively associated with religious health fatalism, indicating that higher fatalistic beliefs were linked to lower adherence. Health literacy demonstrated a weak positive association with religious health fatalism (Table 3).

**Table 3:** Correlations among religious health fatalism, medication adherence, and health literacy among patients with hypertension in – Mardin, Türkiye, 2025 (n = 211).

	Duration of illness	Number of drugs used	Number of children	Medication adherence	Health literacy	Fatalism of religious health
Age	rho = 0.574 p < 0.001*	rho = 0.141 p = 0.041	rho = 0.432 p < 0.001*	rho = –0.071 p = 0.305	rho = 0.114 p = 0.099	rho = 0.149 p = 0.03*
Duration of Illness		rho = 0.096 p = 0.164	rho = 0.435 p < 0.001*	rho = –0.026 p = 0.707	rho = 0.122 p = 0.076	rho = –0.057 p = 0.413
Number of Drugs Used			rho = 0.155 p = 0.024*	rho = –0.213 p = 0.002*	rho = –0.017 p = 0.807	rho = 0.075 p = 0.279
Number of Children				rho = –0.017 p = 0.807	rho = 0.138 p = 0.045*	rho = –0.042 p = 0.544
Medication Adherence					rho = 0.004 p = 0.955	rho = –0.207 p = 0.003*
Health Literacy						rho = 0.167 p = 0.015*

(Correlation is significant at the 0.05 level).

Simple linear regression analyses indicated that religious health fatalism was significantly associated with both medication adherence and health literacy. Higher levels of fatalism were associated with lower medication adherence, indicating a negative relationship. In contrast, a weak but statistically significant positive association was observed between religious health fatalism and health literacy. Health literacy was not significantly associated with medication adherence in regression analysis (Table 4).

**Table 4:** Simple linear regression analyses examining the associations between religious health fatalism, health literacy, and medication adherence among patients with hypertension – Mardin, Türkiye, 2025 (n = 211).

Dependent variables	Independent variables	B	SE	t	p	Confidence interval	
						Min	Max
Health Literacy	Constant	27.910	1.877	14.865		24.208	31.610
	Religious Health Fatalism	0.064	0.031	2.069	0.04*	0.003	0.125
Medication Adherence	Constant	26.587	1.089	24.421		24.440	28.733
	Religious Health Fatalism	-0.073	0.018	-4.091	<0.001*	-0.109	-0.038
	Constant	21.437	1.326	16.156		18.821	24.052
	Health Literacy	0.024	0.041	0.600	0.548	-0.056	0.106

(B = unstandardized regression coefficient; SE = standard error; CI = 95% confidence interval. Separate simple linear regression models were performed for each predictor–outcome pair. Statistical significance was set at p < 0.05).

## DISCUSSION

This study investigated the relationships between religious health fatalism, medication adherence, and health literacy among patients with hypertension. Hypertension constitutes a major global public health concern, particularly in low- and middle-income countries, despite its preventability and treatability^(1)^. According to the World Health Organization (2023), approximately 1.28 billion adults aged 30–79 years are affected by hypertension, with two-thirds residing in low- and middle-income countries^(2)^. In Türkiye, hypertension represents a significant public health problem, with national population-based studies reporting a prevalence of approximately 30–32% among adults^(3,4)^. In the present study, 44.5% of participants reported an income equal to their expenses. Overall, the sociodemographic characteristics and disease prevalence in our sample are consistent with the broader literature.

The mean total score on the Religious Health Fatalism Scale was slightly above the moderate level, indicating a tendency toward fatalistic beliefs among participants. High levels of health fatalism have also been reported in previous studies involving patients with chronic diseases and hypertension^(19,20)^. Similarly, elevated fatalistic beliefs have been documented in individuals with hypertension^(7,21)^. A technical meta-analytic report including 46 studies suggested that stronger fatalistic beliefs were associated with lower engagement in health-promoting behaviors^(22)^. The relatively high level of fatalism observed in this study may be associated with cultural and religious belief systems, particularly the perception that illness is divinely determined^(11)^.

The mean medication adherence score indicated a favorable level of adherence among participants. Similar findings have been reported in previous studies, which documented moderate-to-high levels of medication adherence among patients with hypertension^(23)^. In addition, the majority of participants lived with their spouses and children. However, the potential influence of family support on medication adherence was not directly assessed in this study.

Health literacy levels were found to be moderate among participants. Given the maximum possible score on the health literacy scale, the mean scores suggest that there is room for improvement. Moderate levels of health literacy in hypertensive patients have also been reported in previous studies^(24)^. In addition, limited or insufficient health literacy has also been identified in similar populations^(25,26)^. Furthermore, a high proportion of patients with moderate health literacy levels has been reported in other settings^(27)^. These findings highlight the need for targeted interventions to improve health literacy.

A statistically significant negative association was identified between religious health fatalism and medication adherence (B = –0.073, p < 0.001), indicating that individuals with higher levels of fatalism are less likely to adhere to prescribed treatments. Similar findings have been reported in previous studies, where fatalistic beliefs were associated with lower adherence to treatment^(9,28)^. In cultural contexts where religious beliefs are prominent, fatalism may reduce individuals’ perceived control over their health, thereby decreasing motivation to comply with treatment regimens.

Contrary to H2, the hypothesized negative relationship between religious health fatalism and health literacy was not supported. Instead, a weak but statistically significant positive association was observed (B = 0.064, p = 0.04). This unexpected finding may reflect the cultural context of the study population. In societies where religion plays a central role in daily life, individuals may simultaneously possess greater health-related knowledge and stronger religious beliefs. Therefore, higher health literacy may not necessarily reduce fatalistic beliefs. Rather, religious interpretations of health and illness may coexist with health knowledge, resulting in a positive association between these variables. In the present sample, a considerable proportion of participants had low educational levels, which may limit functional health literacy while still allowing individuals to acquire health-related information through informal or culturally mediated sources. In such contexts, this finding may reflect contextual or sample-specific factors and should be interpreted cautiously. Similarly, previous studies have reported that health literacy levels among individuals with chronic diseases are often limited^(29)^, supporting the notion that literacy may not always translate into effective health behaviours. Therefore, the observed relationship may reflect a culturally embedded form of health understanding, where religious beliefs and health knowledge interact rather than conflict.

Additionally, a weak positive association was found between health literacy and medication adherence (B = 0.024, p = 0.548), although this relationship was not statistically significant in this study. While previous studies have reported that higher health literacy is associated with better adherence to treatment, this finding suggests that the association may be limited or confounded by other factors^(13)^. Similarly, positive associations between treatment adherence and quality of life have been documented^(2)^.

The lack of statistical significance in this study may be partly explained by the high proportion of participants with low educational levels, which may limit their understanding of health-related information. These findings suggest that integrating cultural and religious perspectives into patient education programs may be important for improving treatment adherence in individuals with chronic diseases such as hypertension.

From a nursing perspective, these results highlight the importance of addressing religious health fatalism as part of comprehensive hypertension management. Nurses are in a key position to identify and explore patients’ belief systems through culturally sensitive communication and individualized counseling. By addressing these beliefs, nursing interventions can positively influence patients’ perceptions and behaviors, ultimately improving adherence to treatment^(14,15)^.

In addition, nurses play a crucial role in promoting medication adherence by providing tailored education, reinforcing the importance of consistent medication use, and offering ongoing follow-up^(15)^. Enhancing health literacy is another essential component of nursing practice. Nurses can improve patients’ understanding of their condition and treatment by simplifying complex medical information, using clear communication strategies, and encouraging active participation in decision-making. These approaches contribute to better self-management and improved health outcomes among patients with hypertension^(13,15)^.

### Research Limitations

Several limitations should be considered when interpreting the findings of this study. First, the cross-sectional design precludes any conclusions regarding causal relationships among religious health fatalism, health literacy, and medication adherence. Second, data were collected using self-report instruments, which may be subject to recall bias and social desirability bias. Third, participants were recruited from two public hospitals located within a single region of southeastern Türkiye. Therefore, the findings may not be generalizable to all patients with hypertension or to populations with different cultural and socioeconomic characteristics. Future studies using longitudinal designs and more diverse samples are recommended to further clarify the relationships among these variables.

## CONCLUSION

This study examined the relationships among religious health fatalism, medication adherence, and health literacy in patients with hypertension. The findings demonstrated that higher levels of religious health fatalism were associated with lower medication adherence. Although health literacy showed a weak positive correlation with medication adherence, this association was not statistically significant in regression analysis. These findings suggest that religious health fatalism may play a more prominent role than health literacy in influencing medication adherence among patients with hypertension.

The results highlight the importance of culturally sensitive and evidence-based interventions that address patients’ belief systems in addition to providing health information. Healthcare professionals, particularly nurses, should assess fatalistic beliefs, support medication adherence, and promote health literacy through individualized and patient-centered educational approaches. Future studies using longitudinal and multicenter designs are recommended to further explore these relationships in diverse populations.

## Data Availability

The entire dataset supporting the results of this study is available upon request to the corresponding author.
