# Supplementary material for: The relationship between religious health fatalism, medication adherence, and health literacy among patients with hypertension: a cross-sectional study
Source: Rev Esc Enferm USP. 2026 Aug 7;60:e20250494. doi: 10.1590/1980-220X-REEUSP-2025-0494en (PMC13455963; doi:10.1590/1980-220X-REEUSP-2025-0494en)
Supplement: Translation

# Translation: Relación entre el fatalismo religioso en salud, la adherencia al tratamiento farmacológico y la alfabetización en salud en pacientes con hipertensión: un estudio transversal

**DOI:** 10.1590/1980-220X-REEUSP-2025-0494es

**Authors:** Tanrıverdi Ömer, Gültekin Abdurrezzak

**Affiliations:** 1 Mardin Artuklu University, Faculty of Health Sciences, Department of Nursing, Mardin, Türkiye.; 2 Kahramanmaraş Sütçü İmam University, Afşin School of Health, Department of Nursing, Kahramanmaraş, Türkiye.

**Keywords:** Hipertensión, Alfabetización en Salud, Cumplimiento de la Medicación, Enfermería

## Abstract

**Objetivos:**

La hipertensión continúa siendo uno de los principales desafíos de salud pública a nivel mundial, que requiere una autogestión constante, especialmente mediante la adherencia al tratamiento farmacológico y una adecuada alfabetización sanitaria. Este estudio tuvo como objetivo examinar la relación entre el fatalismo religioso en salud, la adherencia al tratamiento farmacológico y la alfabetización sanitaria en pacientes con hipertensión.

**Métodos:**

Se realizó un estudio descriptivo y transversal con 211 pacientes hipertensos atendidos en dos hospitales públicos del sureste de Turquía. Los datos se recopilaron mediante un cuestionario sociodemográfico, la Escala de Fatalismo Religioso en Salud (RHFS), la Escala de Reporte de Adherencia a la Medicación (MARS) y la Escala de Alfabetización en Salud (HLS).

**Resultados:**

La puntuación media de la RHFS fue de 59,41 ± 12,14, lo que indica un nivel moderado de creencias fatalistas. El fatalismo religioso en salud se correlacionó negativamente con la adherencia al tratamiento farmacológico (r = −0,21; p < 0,001). Se observaron correlaciones positivas débiles entre el fatalismo y la alfabetización sanitaria (r = 0,16; p = 0,04), así como entre la alfabetización sanitaria y la adherencia al tratamiento farmacológico (r = 0,17; p = 0,015).

**Conclusión:**

Aunque la alfabetización sanitaria se asoció positivamente con la adherencia al tratamiento farmacológico en el análisis de correlación, esta asociación no permaneció estadísticamente significativa en el modelo de regresión. El fatalismo religioso en salud mostró una asociación más fuerte con la adherencia al tratamiento farmacológico.

## INTRODUCCIÓN

La hipertensión (HT) es una de las afecciones crónicas más prevalentes en todo el mundo y sigue siendo una importante preocupación de salud pública a pesar de ser en gran medida prevenible y tratable^(1)^. Según la Organización Mundial de la Salud, aproximadamente 1280 millones de adultos de entre 30 y 79 años padecen hipertensión a nivel mundial, y casi dos tercios de ellos viven en países de ingresos bajos y medios^(2)^. La carga de la hipertensión varía según los niveles de ingresos, observándose una mayor prevalencia y menores tasas de conocimiento, tratamiento y control en entornos de ingresos bajos y medios en comparación con los países de ingresos altos^(2)^. Estas disparidades suelen estar asociadas con diferencias en el acceso a la atención médica, la educación y las condiciones socioeconómicas. En Turquía, la hipertensión representa un problema de salud pública significativo, y los estudios nacionales basados en la población reportan una prevalencia de aproximadamente el 30-32% entre los adultos^(3,4)^.

Un desafío importante en el manejo de la hipertensión es la baja adherencia a los regímenes de tratamiento prescritos. Los estudios indican que casi la mitad de las personas diagnosticadas con hipertensión interrumpen el tratamiento durante el primer año, y entre quienes continúan, solo alrededor de la mitad se adhiere de manera consistente a su medicación según lo recomendado^(5)^. Múltiples factores contribuyen a la falta de adherencia, incluyendo el conocimiento limitado, los efectos secundarios de los medicamentos, los costos del tratamiento, el olvido y la naturaleza crónica de la terapia^(6)^.

Además, la naturaleza asintomática de la hipertensión y las creencias de los pacientes respecto a la necesidad de la medicación pueden reducir aún más el cumplimiento. En el contexto de las enfermedades crónicas, las personas suelen recurrir a las creencias religiosas como mecanismo de afrontamiento junto con el tratamiento biomédico^(7)^. El fatalismo en materia de salud, definido como la creencia de que los resultados de salud están predeterminados y fuera del control individual, se ha identificado como un factor importante que influye en los comportamientos de salud^(8)^. Estudios previos han demostrado que los niveles más altos de creencias fatalistas se asocian con una menor participación en comportamientos que promueven la salud y en la adherencia al tratamiento^(8,9)^, y también pueden estar relacionados con niveles más bajos de educación y alfabetización en salud^(10)^. Los factores religiosos y culturales desempeñan un papel significativo en la formación de actitudes fatalistas. En algunos contextos, las interpretaciones religiosas pueden reforzar la percepción de que la enfermedad es inevitable o está predeterminada por la voluntad divina, lo que puede reducir el control que las personas perciben que tienen sobre su salud^(11)^. Además, el fatalismo se ha asociado con la alfabetización en salud. Las personas con una alfabetización en salud limitada pueden ser más propensas a adoptar creencias fatalistas debido a las dificultades para comprender los procesos de la enfermedad y los requisitos del tratamiento^(10)^. Esta interacción entre el fatalismo y la alfabetización en salud puede influir negativamente en la adherencia al tratamiento y en los resultados generales de salud.

Aunque estudios previos han examinado las relaciones entre las creencias fatalistas, la alfabetización en salud y la adherencia a la medicación, estas variables se han investigado generalmente por separado^(8,9,12,13)^. La evidencia sugiere que el fatalismo está asociado con los comportamientos de salud y el autocuidado^(8,9)^, mientras que la alfabetización en salud se ha relacionado con la adherencia al tratamiento y los resultados de salud^(13)^. Sin embargo, son escasas las investigaciones que han explorado cómo el fatalismo religioso en materia de salud, la alfabetización en salud y la adherencia a la medicación interactúan simultáneamente dentro de la misma población, particularmente en contextos con influencias culturales y religiosas. Además, el papel potencial de la alfabetización en salud en la configuración de la relación entre el fatalismo y la adherencia sigue sin estar claro. Abordar esta brecha es importante para desarrollar intervenciones culturalmente sensibles y efectivas destinadas a mejorar el manejo de las enfermedades crónicas. Por lo tanto, este estudio tiene como objetivo examinar la relación entre el fatalismo religioso en materia de salud, la adherencia a la medicación y la alfabetización en salud entre pacientes con hipertensión.

Estos hallazgos pueden tener implicaciones importantes para los sistemas de atención de salud y la práctica de enfermería. Comprender el papel del fatalismo religioso en materia de salud en la configuración de los comportamientos de salud de los pacientes puede apoyar el desarrollo de modelos de atención culturalmente sensibles. Estudios previos han enfatizado que las intervenciones adaptadas culturalmente son efectivas para mejorar el manejo de la hipertensión y la participación de los pacientes en diversas poblaciones^(14)^. En entornos de atención de salud, integrar la evaluación de las creencias fatalistas en las evaluaciones rutinarias de los pacientes puede ayudar a identificar a las personas en riesgo de una mala adherencia a la medicación. Desde una perspectiva de enfermería, estos hallazgos resaltan el papel fundamental de las enfermeras en la prestación de educación individualizada, la mejora de la alfabetización en salud y el abordaje de los sistemas de creencias que pueden obstaculizar el autocontrol efectivo. Las enfermeras contribuyen a mejorar la alfabetización en salud al simplificar la información médica compleja, apoyar la comprensión de los pacientes sobre los regímenes de tratamiento y promover la toma de decisiones informadas^(13)^. A través de estas funciones, las enfermeras pueden ayudar a los pacientes a interpretar mejor la información de salud y a participar más activamente en su proceso de tratamiento^(14,15)^.

Por lo tanto, este estudio tuvo como objetivo examinar la relación entre el fatalismo religioso en materia de salud, la adherencia a la medicación y la alfabetización en salud entre los pacientes con hipertensión.

Este estudio puso a prueba las siguientes hipótesis:

H1: Se espera que el fatalismo religioso en materia de salud se asocie negativamente con la adherencia a la medicación.
H2: Se espera que el fatalismo religioso en materia de salud se asocie negativamente con la alfabetización en salud.
H3: Se espera que la alfabetización en salud se asocie positivamente con la adherencia a la medicación.

### Fundamento

Estudios previos sugieren que las creencias fatalistas pueden reducir la participación de las personas en comportamientos de autocontrol de la enfermedad, incluida la adherencia a la medicación. Además, una alfabetización en salud limitada puede contribuir al pensamiento fatalista e influir negativamente en la adherencia al tratamiento. Basándose en este marco, el presente estudio examinó las relaciones entre el fatalismo religioso en materia de salud, la alfabetización en salud y la adherencia a la medicación en pacientes con hipertensión.

## MÉTODO

### Diseño de la Investigación

Este estudio descriptivo transversal se llevó a cabo entre pacientes con hipertensión.

### Entorno del Estudio

El estudio se llevó a cabo entre pacientes con hipertensión que acudieron a las clínicas ambulatorias de medicina interna de dos hospitales públicos en Mardin, al sureste de Turquía, entre diciembre de 2024 y marzo de 2025. Se incluyeron en el estudio los pacientes que cumplieron con los criterios de elegibilidad y dieron su consentimiento informado.

### Población Y Muestra Del Estudio

El estudio incluyó a pacientes con hipertensión que acudieron a las clínicas ambulatorias de medicina interna entre diciembre de 2024 y marzo de 2025. Se realizó un análisis de poder a priori utilizando G*Power (versión 3.1.9.4) para determinar el tamaño de muestra requerido. Basándose en un tamaño del efecto de 0,30, un nivel de significación de 0,05 y un poder estadístico de 0,95, se calculó que el tamaño mínimo de muestra requerido era de 138 participantes. Sin embargo, el estudio se completó finalmente con un total de 211 participantes.

### Criterios de Inclusión y Exclusión

Los criterios de inclusión fueron los siguientes: pacientes diagnosticados con hipertensión desde hacía al menos 6 meses, de 18 años o más, sin antecedentes de enfermedades psiquiátricas y sin ninguna discapacidad visual, auditiva o del habla que pudiera dificultar la comunicación. Los participantes fueron reclutados entre los pacientes que acudieron a las consultas externas de medicina interna durante el período de estudio, utilizando un enfoque de muestreo consecutivo. Se informó a las personas elegibles sobre el estudio, y aquellas que aceptaron participar fueron incluidas tras proporcionar su consentimiento informado por escrito. Los pacientes que no cumplían los criterios de inclusión, se negaron a participar o tenían datos incompletos o faltantes en el cuestionario fueron excluidos del estudio.

### Formulario Sociodemográfico

El formulario de datos sociodemográficos incluía 10 ítems que abarcaban la edad, el diagnóstico, el género, el estado civil, el nivel educativo, la situación de convivencia, el nivel de ingresos, el lugar de residencia, el hábito de fumar y los antecedentes familiares de hipertensión.

*Escala de fatalismo religioso en la salud* (Religious Health Fatalism Scale – RHFS): La escala fue desarrollada por Franklin et al.^(16)^ para determinar si el fatalismo en la salud en general está relacionado con los comportamientos de salud. El estudio de validez y fiabilidad de la escala en Turquía fue realizado por Bobov y Capik en 2020^(11)^. La escala consta de 17 ítems y una subdimensión. La escala es de tipo Likert de 5 puntos y el rango de puntuación total varía entre 17 y 85. A medida que aumenta la puntuación obtenida en la escala, aumenta el nivel de tendencia al fatalismo. En la escala original, se determinó que el coeficiente de confiabilidad alfa de Cronbach era de 0,91^(11)^. En el presente estudio, se determinó que el coeficiente de confiabilidad alfa de Cronbach era de 0,91.

*Escala de Informe de Adherencia a la Medicación* (Medication Adherence Report Scale-MARS): La Escala de Informe de Adherencia a la Medicación (Medication Adherence Report Scale, MARS) es un instrumento ampliamente utilizado para evaluar la adherencia a la medicación. La versión turca de la escala fue traducida y validada por Temeloğlu Şen et al.^(10)^ en 2019. La MARS consta de 5 ítems y se califica en una escala de Likert de 5 puntos que va de 1 (siempre) a 5 (nunca). La puntuación total se calcula sumando las puntuaciones de los ítems, lo que da un rango posible de 5 a 25. Las puntuaciones más altas indican una mejor adherencia a la medicación, mientras que las puntuaciones más bajas reflejan una menor adherencia. Se informó que la consistencia interna de la versión turca era aceptable, con un alfa de Cronbach de 0,78^(10)^. En el presente estudio, el coeficiente de confiabilidad alfa de Cronbach se calculó en 0,72.

*Escala de Alfabetización en Salud* (Health Literacy Scale-HLS): El estudio de validez y confiabilidad de la adaptación turca de la escala desarrollada originalmente por Suka et al.^(17)^ fue realizado por Türkoğlu y Kılıç en 2021^(18)^. La escala consta de 14 ítems, cada uno calificado en una escala de Likert de cinco puntos que va de 1 (totalmente en desacuerdo) a 5 (totalmente de acuerdo), con puntuaciones totales que oscilan entre 14 y 70. Las puntuaciones más altas indican niveles más altos de alfabetización en salud. La escala comprende tres subdimensiones: Alfabetización en salud funcional (ítems 1–5), Alfabetización en salud interactiva (ítems 6–10) y Alfabetización en salud crítica (ítems 11–14). El coeficiente alfa de Cronbach de la versión turca original fue de 0,85. En el presente estudio, el coeficiente de confiabilidad alfa de Cronbach se calculó en 0,78.

### Análisis Estadístico

Los datos se analizaron utilizando IBM SPSS versión 23. Se utilizaron estadísticas descriptivas, incluyendo número, porcentaje, mínimo, máximo, media y desviación estándar, para resumir los datos. La normalidad de las variables continuas se evaluó mediante la prueba de Kolmogorov-Smirnov. Dado que las puntuaciones de la escala no mostraron una distribución normal, se realizó un análisis de correlación rho de Spearman para examinar las relaciones entre las variables. Además, se realizaron análisis de regresión lineal simple para explorar las asociaciones entre el fatalismo religioso en materia de salud, la adherencia a la medicación y la alfabetización en salud. Antes de los análisis de regresión, se evaluaron supuestos como la linealidad, la independencia de los errores, la homocedasticidad y la normalidad de los residuos, y se determinó que eran satisfactorios. La significación estadística se fijó en p < 0,05.

### Fuentes Potenciales de Sesgo

Es posible que se haya producido un sesgo de selección, ya que los participantes fueron reclutados en dos hospitales públicos ubicados en la misma región geográfica del sureste de Turquía, lo que puede limitar la generalización de los hallazgos a poblaciones más amplias.

### Aprobación Ética

Se obtuvo la aprobación ética del Comité de Ética No Intervencionista de la Universidad Artuklu de Mardin (Decisión n.º: 2024/11-23). También se obtuvo el permiso para realizar el estudio del Hospital de Formación e Investigación de Mardin. Todos los procedimientos se llevaron a cabo de conformidad con la Declaración de Helsinki. Se obtuvo el consentimiento informado por escrito de todos los participantes antes de su inclusión en el estudio.

## RESULTADOS

Los participantes tenían una edad media de 59,86 ± 13,81 años, la duración de la hipertensión (años) era de 8,99 ± 6,96, el 58,3% eran mujeres, el 89,1% estaban casados, el 43,1% vivían en un centro provincial, el 39,82% eran analfabetos, el 44,5% tenía unos ingresos iguales a los gastos, el 66,4% tenía un familiar con hipertensión y el 54,5% no fumaba (Tabla 1).

**Tabla 1:** Características sociodemográficas de los participantes en – Mardin, Turquía, 2025 (n = 211).

	Media ± DE	(mín.–máx.)
Edad	59,86 ± 13,81	(24–100)
Duración de la hipertensión (años)	8,99 ± 6,96	(1–32)
	**Frecuencia**	**Porcentaje**
**Sexo**		
Hombres	88	41,7
Mujer	123	58,3
**Estado civil**		
Casado	188	89,1
Soltero	23	10,9
**Nivel educativo**		
Analfabetos	84	39,8
Alfabetizado	38	18,0
Graduado en educación primaria	50	23,7
Graduado de secundaria	25	11,8
Graduado de secundaria y universidad	14	6,6
**Situación de convivencia**		
Solo	25	11,8
Con cónyuge	46	21,8
Con cónyuge e hijos	135	64,0
Con familiares	5	2,4
**Situación de ingresos**		
Ingresos inferiores a los gastos	92	43,6
Mis ingresos son iguales a mis gastos	94	44,5
Mis ingresos superan mis gastos	25	11,8
**Lugar de residencia**		
Provincia	91	43,1
Distrito	83	39,3
Pueblo	37	17,6
**Consumo de tabaco**		
Sí	96	45,5
No	115	54,5
**Hipertensión en la familia**		
Sí	140	66,4
No	71	33,6

(Media: media; DE: desviación estándar; Mín.: mínimo; Máx.: máximo).

En general, los participantes mostraron niveles moderados de alfabetización en salud y adherencia a la medicación, mientras que las puntuaciones de fatalismo religioso en materia de salud fueron comparativamente altas. Entre las subdimensiones de la alfabetización en salud, la alfabetización interactiva y la funcional fueron similares y ligeramente superiores, mientras que la alfabetización crítica en salud obtuvo la puntuación media más baja, lo que indica un rendimiento comparativamente más débil en este ámbito (Tabla 2).

**Tabla 2:** Estadísticas descriptivas de las puntuaciones de fatalismo religioso en materia de salud, adherencia a la medicación y alfabetización sanitaria de la escala « » en – Mardin, Turquía (n = 211).

	Media ± DE	(mín.–máx.)
Puntuación de fatalismo religioso en materia de salud	59,41 ± 12,14	(21–85)
Puntuación de adherencia a la medicación	22,22 ± 3,27	(10–25)
Puntuación de alfabetización sanitaria	50,36 ± 8,10	(27–70)
Alfabetización funcional en salud	17,58 ± 5,19	(5–25)
Alfabetización sanitaria interactiva	17,90 ± 5,08	(5–25)
Alfabetización sanitaria crítica	14,87 ± 3,49	(4–20)

El análisis de correlación reveló varias relaciones significativas entre las variables. La edad mostró una asociación positiva moderada con la duración de la enfermedad y el número de hijos, lo que indica que las personas de más edad tendían a presentar una mayor duración de la enfermedad y familias más numerosas. El número de medicamentos utilizados se asoció negativamente con la alfabetización en salud, lo que sugiere que las personas con mayor alfabetización en salud tendían a utilizar menos medicamentos. Se observó una relación positiva débil entre el número de medicamentos y el número de hijos. La adherencia a la medicación no mostró ninguna relación significativa con la mayoría de las variables; sin embargo, se asoció negativamente con el fatalismo religioso en materia de salud, lo que indica que unas creencias fatalistas más marcadas se relacionaban con una menor adherencia. La alfabetización en salud mostró una asociación positiva débil con el fatalismo religioso en materia de salud (Tabla 3).

**Tabla 3:** Correlaciones entre el fatalismo religioso en materia de salud, la adherencia a la medicación y la alfabetización en salud entre pacientes con hipertensión en – Mardin, Turquía, 2025 (n = 211).

	Duración de la enfermedad	Número de fármacos utilizados	Número de hijos	Adherencia a la medicación	Alfabetización sanitaria	Fatalismo de la salud religiosa
Edad	rho = 0,574 p < 0,001*	rho = 0,141 p = 0,041	rho = 0,432 p < 0,001*	rho = –0,071 p = 0,305	rho = 0,114 p = 0,099	rho = 0,149 p = 0,03*
Duración de la enfermedad		rho = 0,096 p = 0,164	rho = 0,435 p < 0,001*	rho = –0,026 p = 0,707	rho = 0,122 p = 0,076	rho = –0,057 p = 0,413
Número de fármacos utilizados			rho = 0,155 p = 0,024*	rho = –0,213 p = 0,002*	rho = –0,017 p = 0,807	rho = 0,075 p = 0,279
Número de hijos				rho = –0,017 p = 0,807	rho = 0,138 p = 0,045*	rho = –0,042 p = 0,544
Adherencia al tratamiento					rho = 0,004 p = 0,955	rho = –0,207 p = 0,003*
Alfabetización sanitaria						rho = 0,167 p = 0,015*

(La correlación es significativa al nivel de 0,05).

Los análisis de regresión lineal simple indicaron que el fatalismo religioso en materia de salud se asociaba de manera significativa tanto con la adherencia a la medicación como con la alfabetización en salud. Los niveles más altos de fatalismo se asociaron con una menor adherencia a la medicación, lo que indica una relación negativa. Por el contrario, se observó una asociación positiva débil, pero estadísticamente significativa, entre el fatalismo religioso en materia de salud y la alfabetización en salud. La alfabetización en salud no se asoció de manera significativa con la adherencia a la medicación en el análisis de regresión. (Tabla 4).

**Tabla 4:** Análisis de regresión lineal simple para examinar las asociaciones entre el fatalismo religioso en materia de salud, la alfabetización sanitaria y la adherencia a la medicación entre pacientes con hipertensión en – Mardin, Turquía, 2025 (n = 211).

Variables dependientes	Variables independientes	B	EE	t	p	Intervalo de confianza	
						Mín	Máx
Alfabetización sanitaria	Constante	27.910	1,877	14.865		24,208	31,610
	Salud religiosa Fatalismo	0.064	0.031	2.069	0.04*	0.003	0.125
Adherencia a la medicación	Constante	26,587	1.089	24,421		24.440	28.733
	Salud religiosa Fatalismo	-0.073	0.018	-4.091	<0.001*	-0.109	-0.038
	Constante	21.437	1,326	16,156		18,821	24.052
	Salud religiosa Fatalismo	0.024	0.041	0.600	0.548	-0.056	0.106

(B = coeficiente de regresión no estandarizado; EE = error estándar; IC = intervalo de confianza del 95%. Se realizaron modelos de regresión lineal simple independientes para cada par de variables predictoras y de resultado. La significación estadística se fijó en p < 0,05).

## DISCUSIÓN

Este estudio investigó las relaciones entre el fatalismo religioso en materia de salud, la adherencia a la medicación y la alfabetización en salud entre pacientes con hipertensión. La hipertensión constituye un importante problema de salud pública a nivel mundial, particularmente en los países de ingresos bajos y medios, a pesar de que es prevenible y tratable^(1)^. Según la Organización Mundial de la Salud (2023), aproximadamente 1280 millones de adultos de entre 30 y 79 años padecen hipertensión, y dos tercios de ellos residen en países de ingresos bajos y medios^(2)^. En Turquía, la hipertensión representa un problema de salud pública significativo, y los estudios nacionales basados en la población reportan una prevalencia de aproximadamente el 30–32% entre los adultos^(3,4)^. En el presente estudio, el 44,5% de los participantes reportó unos ingresos iguales a sus gastos. En general, las características sociodemográficas y la prevalencia de la enfermedad en nuestra muestra son consistentes con la literatura más amplia.

La puntuación total media en la Escala de Fatalismo Religioso en Salud se situó ligeramente por encima del nivel moderado, lo que indica una tendencia hacia las creencias fatalistas entre los participantes. También se han descrito altos niveles de fatalismo en salud en estudios previos con pacientes con enfermedades crónicas e hipertensión^(19,20)^. De manera similar, se han documentado creencias fatalistas elevadas en personas con hipertensión^(7,21)^. Un informe metaanalítico técnico que incluyó 46 estudios sugirió que las creencias fatalistas más fuertes se asociaban con una menor participación en comportamientos que promueven la salud^(22)^. El nivel relativamente alto de fatalismo observado en este estudio puede estar asociado con sistemas de creencias culturales y religiosas, particularmente la percepción de que la enfermedad está determinada por la voluntad divina^(11)^.

La puntuación media de adherencia a la medicación indicó un nivel favorable de adherencia entre los participantes. Se han reportado hallazgos similares en estudios previos, que documentaron niveles de adherencia a la medicación de moderados a altos entre pacientes con hipertensión^(23)^. Además, la mayoría de los participantes vivían con sus cónyuges e hijos. Sin embargo, la influencia potencial del apoyo familiar en la adherencia a la medicación no se evaluó directamente en este estudio.

Se observó que los niveles de alfabetización en salud eran moderados entre los participantes. Dada la puntuación máxima posible en la escala de alfabetización en salud, las puntuaciones medias sugieren que hay margen de mejora. En estudios anteriores también se han descrito niveles moderados de alfabetización en salud en pacientes hipertensos^(24)^. Además, también se ha identificado una alfabetización en salud limitada o insuficiente en poblaciones similares^(25,26)^. Además, se ha informado de una alta proporción de pacientes con niveles moderados de alfabetización en salud en otros entornos^(27)^. Estos hallazgos resaltan la necesidad de intervenciones específicas para mejorar la alfabetización en salud.

Se identificó una asociación negativa estadísticamente significativa entre el fatalismo religioso en materia de salud y la adherencia a la medicación (B = −0,073, p < 0,001), lo que indica que las personas con mayores niveles de fatalismo son menos propensas a adherirse a los tratamientos prescritos. Se han reportado hallazgos similares en estudios previos, donde las creencias fatalistas se asociaron con una menor adherencia al tratamiento^(9,28)^. En contextos culturales donde las creencias religiosas son prominentes, el fatalismo puede reducir el control percibido de las personas sobre su salud, disminuyendo así la motivación para cumplir con los regímenes de tratamiento.

Contrariamente a la hipótesis H2, no se confirmó la relación negativa hipotética entre el fatalismo religioso en materia de salud y la alfabetización en salud. En cambio, se observó una asociación positiva débil pero estadísticamente significativa (B = 0,064, p = 0,04). Este hallazgo inesperado puede reflejar el contexto cultural de la población del estudio. En sociedades donde la religión desempeña un papel central en la vida cotidiana, las personas pueden poseer simultáneamente un mayor conocimiento relacionado con la salud y creencias religiosas más sólidas. Por lo tanto, una mayor alfabetización en salud puede no reducir necesariamente las creencias fatalistas. Más bien, las interpretaciones religiosas de la salud y la enfermedad pueden coexistir con el conocimiento sobre la salud, lo que da lugar a una asociación positiva entre estas variables. En la presente muestra, una proporción considerable de participantes tenía bajos niveles educativos, lo que puede limitar la alfabetización funcional en salud, al tiempo que permite a las personas adquirir información relacionada con la salud a través de fuentes informales o mediadas culturalmente. En tales contextos, este hallazgo puede reflejar factores contextuales o específicos de la muestra y debe interpretarse con cautela. De manera similar, estudios previos han informado que los niveles de alfabetización en salud entre las personas con enfermedades crónicas suelen ser limitados^(29)^, lo que respalda la idea de que la alfabetización no siempre se traduce en comportamientos de salud efectivos. Por lo tanto, la relación observada puede reflejar una forma de comprensión de la salud arraigada culturalmente, en la que las creencias religiosas y el conocimiento sobre la salud interactúan en lugar de entrar en conflicto.

Además, se encontró una asociación positiva débil entre la alfabetización en salud y la adherencia a la medicación (B = 0,024, p = 0,548), aunque esta relación no fue estadísticamente significativa en este estudio. Si bien estudios previos han informado que una mayor alfabetización en salud se asocia con una mejor adherencia al tratamiento, este hallazgo sugiere que la asociación puede ser limitada o estar confundida por otros factores^(13)^. De manera similar, se han documentado asociaciones positivas entre la adherencia al tratamiento y la calidad de vida^(29)^.

La falta de significación estadística en este estudio puede explicarse en parte por la alta proporción de participantes con bajos niveles educativos, lo que puede limitar su comprensión de la información relacionada con la salud. Estos hallazgos sugieren que integrar perspectivas culturales y religiosas en los programas de educación para pacientes puede ser importante para mejorar la adherencia al tratamiento en personas con enfermedades crónicas como la hipertensión.

Desde una perspectiva de enfermería, estos resultados resaltan la importancia de abordar el fatalismo religioso en materia de salud como parte del manejo integral de la hipertensión. Las enfermeras se encuentran en una posición clave para identificar y explorar los sistemas de creencias de los pacientes a través de una comunicación culturalmente sensible y un asesoramiento individualizado. Al abordar estas creencias, las intervenciones de enfermería pueden influir positivamente en las percepciones y los comportamientos de los pacientes, mejorando en última instancia la adherencia al tratamiento^(14,15)^.

Además, las enfermeras desempeñan un papel crucial en la promoción del cumplimiento del tratamiento farmacológico al brindar educación personalizada, reforzar la importancia del uso constante de los medicamentos y ofrecer un seguimiento continuo^(15)^. Mejorar la alfabetización en salud es otro componente esencial de la práctica de enfermería. Las enfermeras pueden mejorar la comprensión que tienen los pacientes de su afección y tratamiento al simplificar la información médica compleja, utilizar estrategias de comunicación claras y fomentar la participación activa en la toma de decisiones. Estos enfoques contribuyen a una mejor autocontrol y a mejores resultados de salud entre los pacientes con hipertensión^(13,15)^.

### Limitaciones de la Investigación

Se deben tener en cuenta varias limitaciones al interpretar los hallazgos de este estudio. En primer lugar, el diseño transversal impide sacar conclusiones sobre las relaciones causales entre el fatalismo religioso en materia de salud, la alfabetización en salud y la adherencia a la medicación. En segundo lugar, los datos se recolectaron mediante instrumentos de autoinforme, lo que puede estar sujeto a sesgos de recuerdo y de deseabilidad social. En tercer lugar, los participantes fueron reclutados en dos hospitales públicos ubicados en una sola región del sureste de Turquía. Por lo tanto, es posible que los hallazgos no sean generalizables a todos los pacientes con hipertensión o a poblaciones con características culturales y socioeconómicas diferentes. Se recomiendan estudios futuros que utilicen diseños longitudinales y muestras más diversas para aclarar aún más las relaciones entre estas variables.

## CONCLUSIÓN

Este estudio examinó las relaciones entre el fatalismo religioso en materia de salud, la adherencia a la medicación y la alfabetización en salud en pacientes con hipertensión. Los resultados demostraron que los niveles más altos de fatalismo religioso en materia de salud se asociaban con una menor adherencia a la medicación. Aunque la alfabetización en salud mostró una correlación positiva débil con la adherencia a la medicación, esta asociación no fue estadísticamente significativa en el análisis de regresión. Estos hallazgos sugieren que el fatalismo religioso en materia de salud puede desempeñar un papel más destacado que la alfabetización en salud a la hora de influir en la adherencia a la medicación entre los pacientes con hipertensión.

Los resultados resaltan la importancia de intervenciones culturalmente sensibles y basadas en la evidencia que aborden los sistemas de creencias de los pacientes, además de proporcionar información de salud. Los profesionales de la salud, particularmente las enfermeras, deben evaluar las creencias fatalistas, apoyar la adherencia a la medicación y promover la alfabetización en salud a través de enfoques educativos individualizados y centrados en el paciente. Se recomiendan estudios futuros con diseños longitudinales y multicéntricos para explorar más a fondo estas relaciones en poblaciones diversas.

## Data Availability

El conjunto de datos que respaldan las conclusiones de este estudio está disponible previa solicitud al autor correspondiente.
